# The Metabolic and Endocrine Effects of a 12-Week Allulose-Rich Diet

**DOI:** 10.3390/nu16121821

**Published:** 2024-06-10

**Authors:** Kevin B. Cayabyab, Marley J. Shin, Micah S. Heimuli, Iris J. Kim, Dominic P. D’Agostino, Richard J. Johnson, Andrew P. Koutnik, Nick Bellissimo, David M. Diamond, Nicholas G. Norwitz, Juan A. Arroyo, Paul R. Reynolds, Benjamin T. Bikman

**Affiliations:** 1 Department of Cell Biology and Physiology, Brigham Young University, Provo, UT 84602, USA; 2 Department of Molecular Pharmacology and Physiology, University of South Florida, Tampa, FL 33602, USA; 3 Department of Medicine, University of Colorado, Aurora, CO 80045, USA; 4 Sansum Diabetes Research Institute, Santa Barbara, CA 93105, USA; 5 School of Nutrition, Toronto Metropolitan University, Toronto, ON M5S 1A8, Canada; 6 Department of Psychology, University of South Florida, Tampa, FL 33602, USA; 7 Harvard Medical School, Boston, MA 02115, USA

**Keywords:** insulin resistance, diabetes, obesity, mitochondria, allulose

## Abstract

The global rise in type 2 diabetes (T2D) and obesity necessitates innovative dietary interventions. This study investigates the effects of allulose, a rare sugar shown to reduce blood glucose, in a rat model of diet-induced obesity and T2D. Over 12 weeks, we hypothesized that allulose supplementation would improve body weight, insulin sensitivity, and glycemic control. Our results showed that allulose mitigated the adverse effects of high-fat, high-sugar diets, including reduced body weight gain and improved insulin resistance. The allulose group exhibited lower food consumption and increased levels of glucagon-like peptide-1 (GLP-1), enhancing glucose regulation and appetite control. Additionally, allulose prevented liver triglyceride accumulation and promoted mitochondrial uncoupling in adipose tissue. These findings suggest that allulose supplementation can improve metabolic health markers, making it a promising dietary component for managing obesity and T2D. Further research is needed to explore the long-term benefits and mechanisms of allulose in metabolic disease prevention and management. This study supports the potential of allulose as a safe and effective intervention for improving metabolic health in the context of dietary excess.

## 1. Introduction

The escalating global prevalence of type 2 diabetes (T2D) and obesity represents a significant public health challenge, necessitating the exploration of innovative dietary strategies to mitigate these conditions. The burgeoning epidemic of type 2 diabetes (T2D) and obesity worldwide requires a relentless pursuit of innovative and effective interventions.

Allulose (d-psicose) is a rare natural sugar found naturally in small quantities in certain fruits. Allulose offers the sweetness of fructose yet is metabolically distinct; it is the C-3 epimer of fructose that, unlike fructose, has no effect on glucose or insulin. Furthermore, unlike fructose, allulose is not a substrate for de novo lipogenesis and elicits no inhibitory effect on fatty acid oxidation [1]. Altogether, these reasons make allulose an attractive candidate for the dietary management of metabolic disorders, both by displacing dietary fructose as well as providing putative metabolic benefits itself [1,2,3]. Unlike traditional sugars, allulose is largely absorbed by the small intestine and excreted without being fully metabolized [4], thus providing a low-calorie alternative to sucrose and high-fructose corn syrup, in addition to other low- or zero-calorie sweeteners [5].

Recent studies in humans have highlighted allulose’s potential benefits, including improved glycemic control and reduced adiposity, without the deleterious metabolic effects associated with conventional sugars [1,6,7,8]. However, while clearly eliciting an increase in endogenous glucagon-like peptide-1 (GLP-1) release [9], which is heavily exploited with weight loss drugs [10,11], the mechanisms underlying these beneficial effects remain to be fully elucidated. Furthermore, while human studies provide critical insights, animal models, particularly rodent models of T2D and obesity, offer valuable opportunities to explore the physiological, metabolic, and molecular responses to allulose in a controlled setting.

This 12-week study aimed to investigate the effects of dietary allulose supplementation in a rat model of diet-induced obesity and T2D. We hypothesized that allulose supplementation would lead to significant improvements in body weight, insulin sensitivity, and glycemic control compared to control animals fed a standard diet without allulose. Additionally, we sought to explore the potential mechanisms by which allulose exerts its effects, including its impact on adipocyte metabolism and adipokine production, liver nutrient handling, and inflammatory markers.

By elucidating the metabolic effects of allulose in a well-established animal model of T2D and obesity, this study seeks to contribute to the growing body of literature supporting the use of allulose as a safe and effective dietary intervention for managing these conditions. The findings from this research could have significant implications for dietary recommendations, food industry practices, and the development of novel therapeutic strategies targeting the etiologies of T2D and obesity. The aim of this study was to confirm previous findings on the effect of allulose in mitigating some of the metabolic effects of a Western diet and add to this by including outcomes specific to adipose physiology and mitochondrial bioenergetics.

## 2. Materials and Methods

### 2.1. Animals

Twelve-week-old female and male Wistar rats were purchased from Jackson Laboratories (Bar Harbor, ME, USA) and housed at 22 ± 1 °C on a 12 h light–dark cycle. Animals were randomly divided into four groups and housed separately (*n* = 10; 5 female, 5 male) for a 12-week trial as follows: standard lab chow with stevia (Stevita Naturals, Kennendale, TX, USA), Western diet chow with stevia, standard lab chow with allulose, or Western diet chow with allulose. Allulose (allSWEET^®^) was provided by Anderson Advanced Ingredients (Irvine, CA, USA). Animals had free access to food and water. The Western diet was Research Diets D12266B (New Brunswick, NJ, USA) which contains sucrose, saturated fat (butter), and polyunsaturated fats (corn oil). Stevia and allulose were provided in drinking water, and each animal had access to 30 ml of sterile water daily with either 0.1 mL of stevia (sweetened to match allulose) or 3% allulose (to reach a daily dose of roughly 1.9 g/kg/day), as has been performed previously [12]. Throughout the protocol, tail vein blood was obtained every four weeks following an 8 h fast for ongoing hormone (insulin and GLP-1) and glucose analysis. Following the 12 wk protocol, animals were used for tolerance tests and sacrificed for tissue extraction for further analysis (outlined below). All work was approved by the Institutional Animal Care and Use Committee (IACUC; 23-1222) at Brigham Young University and conducted in accordance with the procedures and regulations outlined in the National Institutes of Health Guide for the Care and Use of Laboratory Animals.

### 2.2. ATP Quantification

The ATP concentration (*n* = 8 for each group) was measured using an ATPlite Luminescence Assay kit (Perkin Elmer; Waltham, MA, USA). Tissues were homogenized in ATP stabilization buffer (three volumes of ice-cold PBS containing 20 mM glycine, 50 mM MgSO_4_, and 4 mM EDTA). Homogenates were then diluted at a 1 to 5 ratio in ddH_2_O. A total of 100 μL of ATP stabilizing buffer and 50 μL of mammalian cell lysis buffer were added to an opaque, white, 96-well plate in wells used for standards and were then placed on a shaker for 5 min at 25 °C and 700 RPM. Next, 100 μL of the diluted samples were added into open wells, and 10 μL of the standards were added to the aforementioned ATP stabilizing buffer. Then the dish was placed on the shaker for 5 min at 25 °C and 700 RPM. Next, 50 μL of the substrate was added into each well (in the dark) and was placed on a shaker for 5 min at 25 °C and 700 RPM. Solutions in the wells were allowed to dark adapt for 10 min. The luminescence was subsequently measured using a Victor Nivo Multimode Plate Reader (Perkin Elmer), and the ATP concentration was normalized to the protein concentration measured by a BCA protein assay (Thermo Fisher Scientific; Waltham, MA, USA).

### 2.3. Mitochondrial Respiration

Mitochondrial oxygen consumption rates were determined at 37 °C from freshly isolated tissue using the Oroboros O2K Oxygraph (Oroboros, Innsbruck, Austria) with MiR05 respiration buffer as described previously [13]. Before the adipose samples were transferred to the respirometer chambers of the oxygraph, the samples were permeabilized in 0.05 mg/mL of saponin (Sigma-Aldrich, St. Louise, MO, USA) in MiR05 for 30 min at 4 °C. After the addition of the permeabilized tissue, the respirometer chambers were hyperoxygenated to ~350 nmol/mL. Oxygen consumption rates were determined with the following protocol: The electron flow through complex I and II was supported by the addition of glutamate + malate + succinate (10 mM, 2 mM, and 10 mM, respectively) to determine the leak oxygen consumption (GMS). Following stabilization, adenosine diphosphate (ADP) (2.5 mM) was added to determine the oxygen consumption associated with oxidative phosphorylation. Following the conclusion of the respiration protocol, the samples were collected from the chambers and stored at −20 °C for further analysis. Protein concentrations were measured via a BCA assay (Perkin Elmer), and the respiration rates were normalized to the protein concentration.

### 2.4. Plasma Protein Analysis

Protein isolation was performed by homogenization with RIPA buffer containing protease inhibitors (Fisher Scientific). Total protein was quantified using a BCA Protein Assay Kit (Fisher Scientific) and 20 µg of protein was used for active immunoblotting or characterization of proteins. Samples were added to membranes from custom rodent antibody arrays (Abcam, Cambridge, UK) containing specific capture antibodies for C-reactive protein, active GLP-1, insulin, adiponectin, and leptin, and allowed to incubate overnight before being retrieved and incubated again with a second antibody array membrane. Biotinylated antibodies were then added to each membrane and incubated overnight followed by a final incubation with a streptavidin-conjugated fluorescent label to detect the cytokine expression. Membranes were imaged using the fluorescence imaging system previously mentioned (LI-COR; Lincoln, NE, USA), and then quantified using Image J version 1.54h (U.S. National Institutes of Health, Bethesda, MD, USA). Signal intensities were compared to positive controls included on each membrane, as we have performed previously [14].

### 2.5. Tolerance Tests

The tolerance testing was divided into three main procedures conducted on separate days with at least a 48 h recovery period between tests: the Pyruvate Tolerance Test (PTT), Glucose Tolerance Test (GTT), and Insulin Tolerance Test (ITT). For each test, blood samples were collected from the tail vein at 0 (baseline), 15, 30, 60, and 120 min post-injection to measure blood glucose levels using a handheld glucometer (Bayer Contour; Whippany, NJ, USA). Following an 8 h fast, rats received an intraperitoneal injection of sodium pyruvate (2 g/kg body weight) for the measurement of plasma pyruvate levels using an enzymatic assay. One week after the PTT, the GTT was performed with a similar fasting period followed by an intraperitoneal injection of glucose (2 g/kg body weight). The ITT was conducted one week following the GTT, and rats were injected intraperitoneally with insulin (0.75 U/kg).

### 2.6. Liver Analysis

Liver tissue samples were collected post-euthanasia, promptly weighed, and homogenized in ice-cold buffer for the determination of triglyceride and glycogen content. Liver triglycerides were quantified using a triglyceride assay kit (ab65336; Abcam, Cambridge, UK), according to the manufacturer’s protocol. Glycogen levels were assessed using a liver glycogen assay kit (ab169558; Abcam). Triglyceride and glycogen content was normalized to liver wet weight. Plasma levels of alanine aminotransferase (ALT) and aspartate aminotransferase (AST) were measured to assess liver function. Blood samples were collected via cardiac puncture immediately following euthanasia, and plasma was obtained by centrifugation at 3000 g for 10 min. Plasma ALT and AST activities were determined using commercially available assay kits (ab263883 and ab285264; Abcam).

### 2.7. Statistical Analysis

Mean values ± S.E.M were assessed by one-way (for single-time measurements) or two-way (for measurements over time) ANOVA, followed by student *t* tests. Results are representative and those with *p* values < 0.05 were considered significant. Statistical analysis was performed with GraphPad Prism 7.0.

## 3. Results

**Figure 1:**
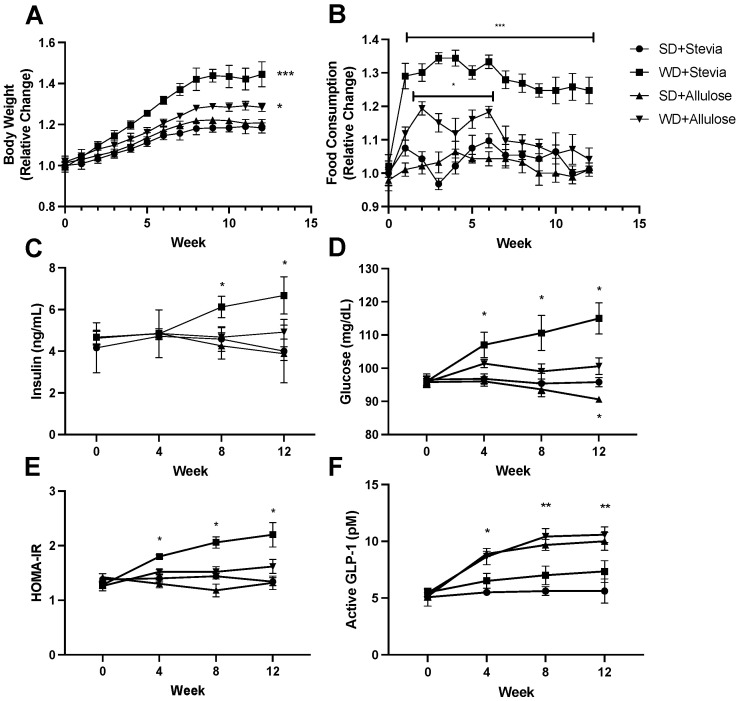
Body weight, food consumption, and endocrine changes in 12 weeks of allulose consumption. Throughout the 12 wk trial, rats manifested several significant changes. Firstly, allulose consumption blunted weight gain with a Western diet ((**A**); *n* = 10), reflected in a reduced overall food consumption ((**B**); *n* = 10). Insulin (**C**) and glucose (**D**) levels, used to compute the HOMA-IR (**E**), while increased significantly in the WD+stevia group, were normal in the WD+allulose group (*n* = 8). GLP-1 was significantly elevated throughout the trial in both groups consuming allulose ((**F**); *n* = 8). * *p* < 0.05; ** *p* < 0.005; *** *p* < 0.0005.

One of the central outcomes of this study was to understand the influence of allulose on altering body weight changes and food consumption, including adding to the mediating mechanisms of such changes. Body weight (Figure 1A; male average starting mass: 328 ± 17 g; female average starting mass: 245 ± 12 g) increased significantly in animals given a Western diet (WD) with stevia. However, the weight gain was significantly less in the WD+allulose groups compared to the WD+stevia group. Importantly, no difference in weight was observed between the rats on stevia or allulose that were receiving the standard diet (SD), suggesting that the allulose effects were specific to a Western diet. Consistent with these findings, there was a marked increase in food intake in the WD+stevia group compared to the SD+stevia group (Figure 1B; male average starting intake: 21 ± 3 g; female average starting intake: 16 ± 2 g). Again, the WD+allulose group showed a significant reduction in food intake compared to the WD+stevia group.

Consistent with the effects on weight, allulose also blocked the development of hyperinsulinemia (Figure 1C), hyperglycemia (Figure 1D), and insulin resistance (via the Homeostatic Model Assessment for Insulin Resistance (HOMA-IR) index; Figure 1E) with a Western diet that was observed in the WD+stevia group. Of interest, the protection with allulose was associated with higher serum levels of the active glucagon-like peptide-1 (GLP-1; Figure 1F). Of note, the higher GLP-1 levels were observed in both allulose groups, but the benefit on weight and metabolic parameters was only observed in the WD+allulose group.

**Figure 2:**
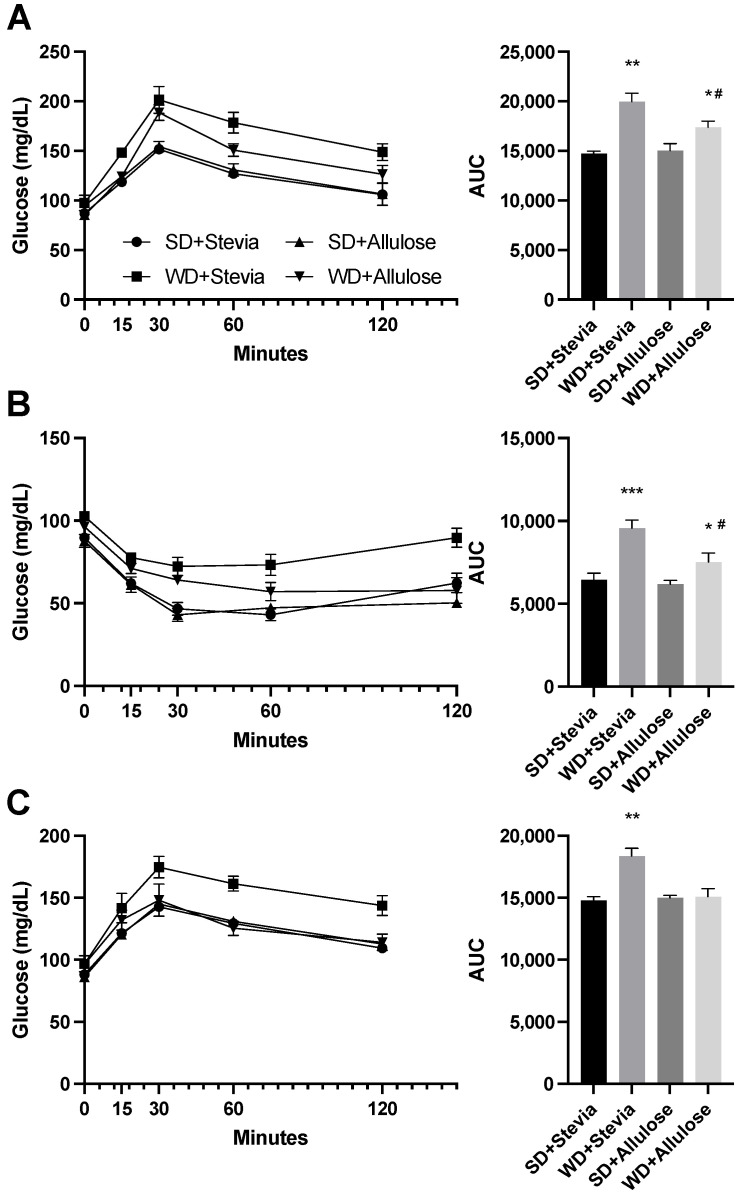
Glucose, insulin, and pyruvate tolerance tests in allulose-fed rats. At the end of the 12 wks trial, animals underwent tolerance tests to glucose ((**A**); 2 g/kg BW), insulin ((**B**); 0.75 IU/kg BW), and pyruvate ((**C**); 0.75 IU/kg BW) (*n* = 6). Based on the area under the curve (AUC), rats on WD+stevia had the most dramatic response to each stimulus, with a blunted response seen in the WD+allulose in glucose (**A**) and insulin (**B**) tolerance tests, and a normal response to pyruvate (**C**). * *p* < 0.05; ** *p* < 0.005; *** *p* < 0.0005 vs. SD+stevia; # *p* < 0.05 vs. WD+stevia.

To further understand the metabolic impact of allulose on whole-body metabolism, we conducted a series of tolerance tests, namely glucose, insulin, and pyruvate (Figure 2A–C). When challenged with an intraperitoneal glucose load (Figure 2A), the WD+stevia group had the greatest glucose response, maintaining a significantly elevated response throughout the test compared with the other groups (consistent with worse insulin resistance). In contrast, the WD+allulose group elicited a moderate response. A similar result was found with the insulin tolerance test (Figure 2B). We also determined whether the Western diet might aggravate gluconeogenesis by performing the pyruvate tolerance test. As expected, the WD+stevia group showed a marked stimulation of gluconeogenesis with a pronounced rise of glucose levels in the serum in response to pyruvate. Of note, gluconeogenesis was blocked by allulose in rats receiving a Western diet (Figure 2C).

**Figure 3:**
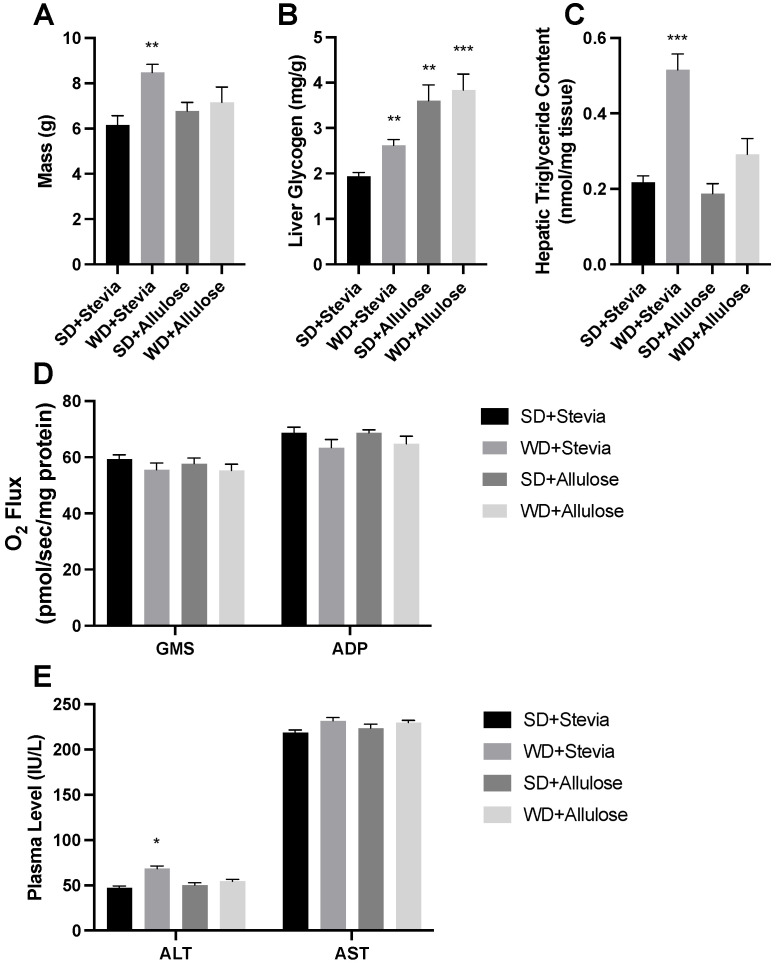
Liver analysis following 12 wks of allulose consumption. Liver mass was measured (**A**) prior to the analysis of glycogen (**B**) and triglycerides (**C**) following 12 wks of a Western diet (WD) and standard diet (SD) with stevia or allulose in the drinking water. Mitochondrial respiration (see Section 2) was measured to determine any direct impact of diet on function (**D**). The liver enzymes alanine (ALT) and aspartate aminotransferase (AST) were quantified (**E**) in the plasma as a marker of liver health. N = 8. * *p* < 0.05; ** *p* < 0.005; *** *p* < 0.0005 vs. SD+stevia.

A primary tissue of interest for allulose is the liver and its differential handling of nutrients, particularly glycogen and hepatic triglycerides. Allulose has been reported to increase the glycogen content in the liver of laboratory rats in association with some increase in mass, despite no effects on liver function or histology [15]. Consistent with that report, we did observe a significant increase in liver size and glycogen content in the animals on standard diets that received allulose compared to those receiving stevia (Figure 3A,B). A Western diet is known to increase both glycogen and fat in the liver, and, as expected, both glycogen and liver fat (triglycerides) were markedly elevated in the WD+stevia group (Figure 3A–C). However, the WD+allulose group, while still showing high glycogen content, was largely protected from the development of fatty liver, and also developed less hepatic hypertrophy (weight gain) compared to the WD+stevia group (Figure 3A–C). Indeed, elevations in liver function tests were only observed in the WD+stevia group (Figure 3E). These studies demonstrate that allulose has a protective effect on hepatic outcomes derivative of a Western diet. Across all groups, liver mitochondrial respiration was unchanged (Figure 3D).

**Figure 4:**
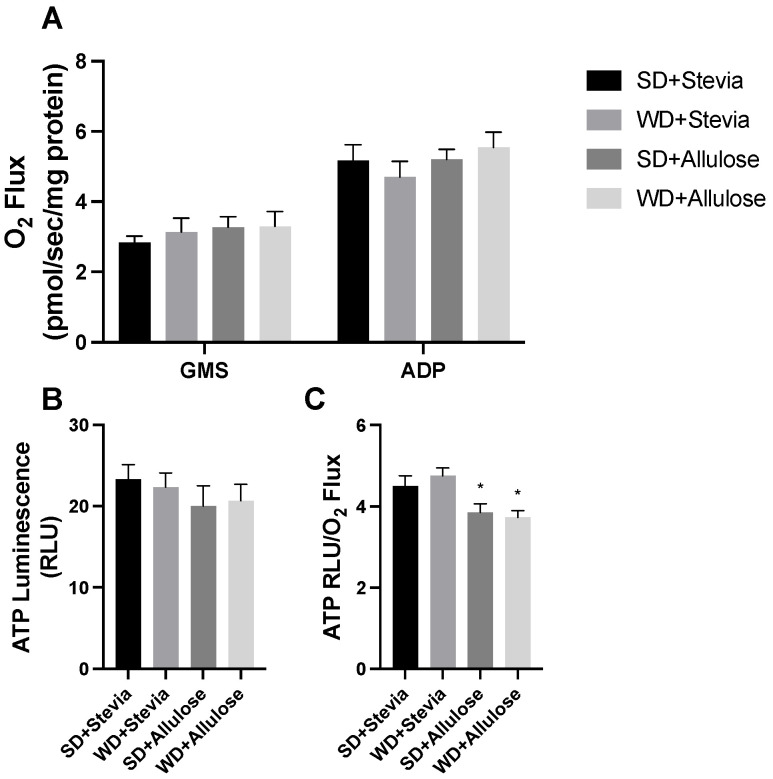
Adipose mitochondrial function analysis following 12 wks of allulose consumption. Mitochondrial respiration ((**A**); see Section 2) was measured in subcutaneous adipose tissue in rats following 12 wks of a Western diet (WD) and standard diet (SD) with stevia or allulose in the drinking water. ATP was also determined (**B**) and a combination of variables was used to determine the degree to which tissue produced ATP based on respiration rates (**C**). N = 7. * *p* < 0.05 vs. SD+stevia.

Due to the changes in body weight, we sought to further understand the bioenergetics within the adipose tissue. We have found previously that various dietary interventions are capable of increasing adipose metabolic rate [16]. While we did not observe any differences across groups in mitochondrial respiration (Figure 4A) or ATP levels (Figure 4B), we did see that the allulose groups had a significantly lower ratio of respiration to ATP levels (Figure 4C), suggesting greater mitochondrial uncoupling.

**Figure 5:**
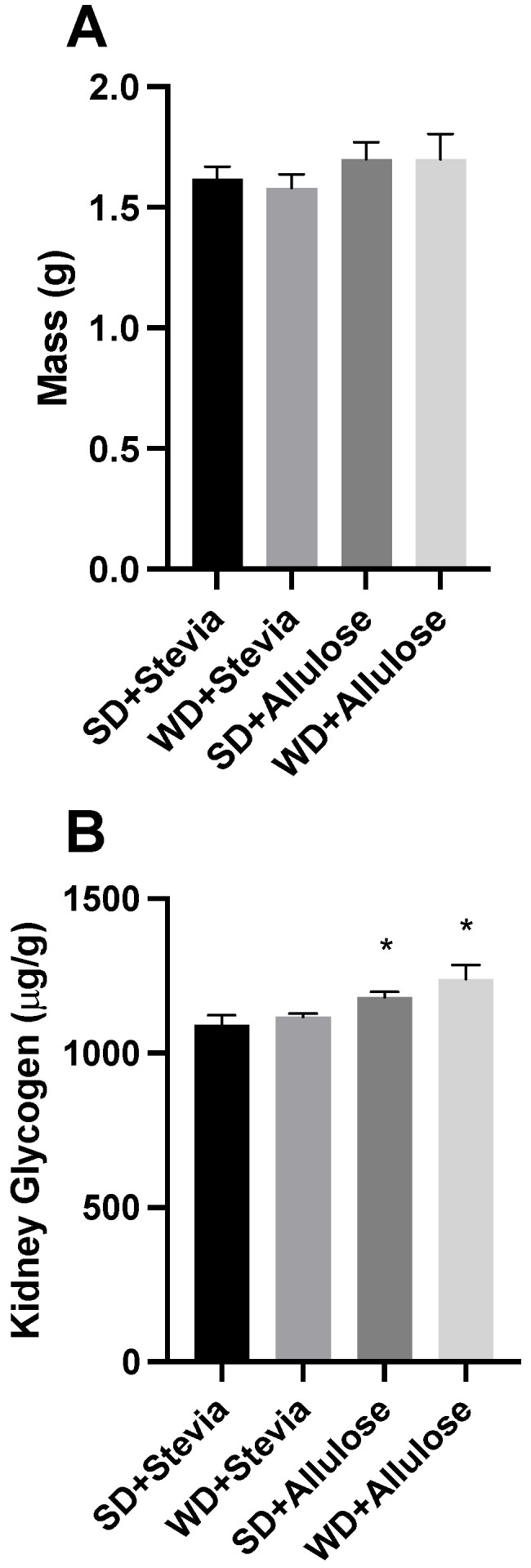
Kidney analysis following 12 wks of allulose consumption. Kidney mass was measured (**A**) prior to analysis of glycogen (**B**) following 12 wks of a Western diet (WD) and standard diet (SD) with stevia or allulose in the drinking water. N = 10. * *p* < 0.05 vs. SD+stevia.

Like the liver, the kidney appears to respond uniquely to allulose. Following the 12 wk trial, we analyzed kidney mass (Figure 5A) and glycogen levels (Figure 5B). While no differences in kidney mass were noted across all groups, there was a slight, yet significant difference in kidney glycogen levels in both allulose groups.

**Figure 6:**
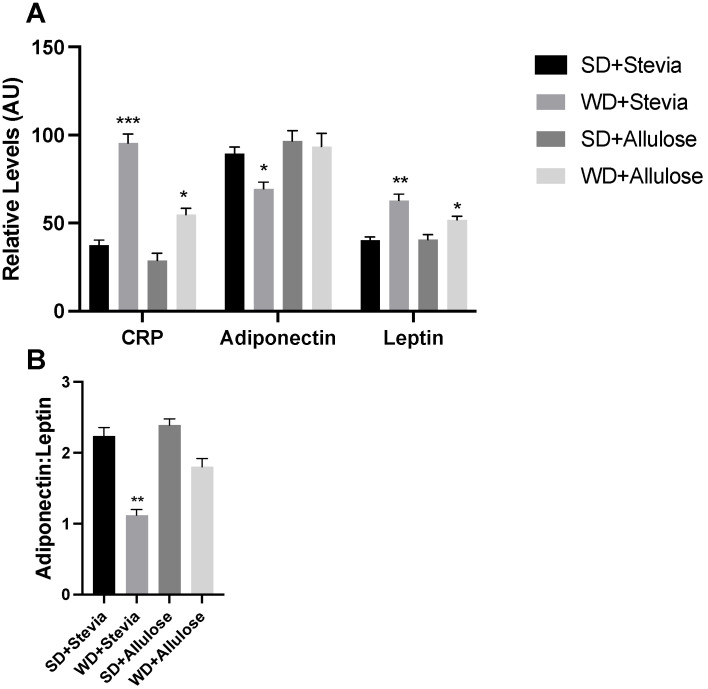
Hormone levels in allulose-fed rats. At the end of the 12 wk trial, plasma was collected from rodents on a Western diet (WD) and standard diet (SD) with stevia or allulose in the drinking water. Analytes included c-reactive protein (CRP), adiponectin, and leptin (**A**). Adiponectin and leptin were further used to create a ratio that is reflective of adipocyte size (**B**). N = 6. * *p* < 0.05; ** *p* < 0.005; *** *p* < 0.0005 vs. SD+stevia.

To gain a deeper understanding of the effects of the intervention on the general inflammatory and metabolic status, we measured levels of several metabolic markers, including c-reactive protein (CRP), adiponectin, and leptin. Animals in the WD+stevia group had an over twofold increase in CRP levels compared with both SD groups (Figure 6A), an effect that was blunted in the WD+allulose group. Adiponectin was significantly reduced in the WD+stevia group only, while leptin generally followed a similar trend, albeit more modest, as seen with CRP. The adiponectin/leptin ratio, a surrogate of adipocyte size [17], revealed a significant reduction in WD+stevia only (Figure 6B).

## 4. Discussion

The outcomes of this study contribute to our understanding of the metabolic effects of allulose, especially in the context of diet-induced changes in body weight, food consumption, insulin resistance, and tissue-specific metabolic responses. Notably, our findings support previous work finding that supplementation with allulose in a Western diet (WD) mitigates some of the adverse effects commonly associated with high-fat, high-sugar diets, such as increased body weight and insulin resistance, findings that bear obvious significance throughout the world [18]. In addition, this work provides novel insight into the GLP-1 dynamics, as well as insight into adipocyte changes and mitochondrial function.

The initial observations of our work act to confirm those found in prior work. The observation that body weight was significantly increased in both WD+stevia and WD+allulose groups compared to their standard diet (SD) counterparts, albeit to different extents, underscores the complex interplay between dietary sugars and weight regulation. The lesser degree of weight gain in the WD+allulose group suggests a potential protective effect of allulose against diet-induced obesity. This is supported by previous research indicating that allulose has anti-obesity effects in both animal and human studies, primarily through a reduction in visceral fat and the improvement of lipid metabolism [5,19,20].

Furthermore, the blunted increase in food consumption observed in the WD+allulose group compared to the WD+stevia group may indicate an appetite-regulating effect of allulose. This finding aligns with studies suggesting that allulose can influence the satiety cascade and reduce overall caloric intake [21]. The significant increase in active glucagon-like peptide-1 (GLP-1) levels in the allulose groups highlights another mechanism through which allulose may exert its metabolic benefits. GLP-1, a hormone involved in glucose regulation and appetite control, has been shown to be elevated by allulose intake, enhancing glucose tolerance and reducing weight gain [9,22]. While others have shown increased GLP-1 secretion in response to allulose [23], we believe our results are the first to report an increased GLP-1 in a state, suggesting that allulose elicits a lingering effect, well past the point of consumption.

The differential responses in insulin and glucose levels between the groups, with the WD+stevia group exhibiting a gradual increase while the WD+allulose group did not, further emphasizes the beneficial role of allulose in modulating glucose homeostasis and insulin sensitivity. These data are consistent with the notion that allulose could improve glycemic control through both glucose deposition and production, improving insulin sensitivity to augment deposition into muscle and reducing production from glycogenosis [1,20], as well as positionally decreasing enteral absorption through substrate competition [18,24].

Our analysis of liver and adipose tissue further elucidates the tissue-specific metabolic effects of allulose, including novel findings of changes in mitochondrial function. While liver triglycerides were only elevated in the WD+stevia group, suggesting that allulose may prevent liver fat accumulation, the significant glycogen accumulation across all intervention groups, particularly in the allulose groups, could reflect a shift in nutrient utilization or storage mechanisms. This finding may partly explain those observed by Liu et al. [15] where they reported an improvement in exercise capacity in allulose-fed rodents.

Given our and others’ findings of protection against obesity with the inclusion of allulose, we sought to leverage our previous experience with analyzing adipose mitochondrial bioenergetics to determine the relevance of allulose in this [13,25]. We observed greater mitochondrial uncoupling in adipose tissue of both allulose groups, suggesting a novel effect of allulose in this process. This mechanism may help explain the observed weight regulation effects. Due to tissue limitations, we were unable to directly measure uncoupling protein levels in adipose tissue to confirm a protein-level change.

This study includes some limitations. One limitation of this study is the use of stevia as a control. We sought to use a control sweetener that is both common and mostly inert. Because of their widely varied natures, it is impossible to be certain that the doses of allulose and stevia used are “equal”. Accordingly, we used doses of both that have been used previously [12,26]. Importantly, while stevia has been shown to stimulate GLP-1 secretion in cell culture, we know of no such evidence in rodents or humans. This suggests that some of the advantages seen with allulose in this study could be specific to GLP-1 release. Furthermore, stevia is an ideal control in this study given previous evidence suggesting that stevia has no effect, compared with water, on metrics of weight gain [27]. However, the lack of a water-only group should be noted. A second limitation is the lack of histological data on the liver and kidney. While our findings suggest a benign effect of allulose supplementation based on kidney and liver nutrient storage and, in the case of liver, enzymes, histological comparisons would have allowed greater insight into the state of these tissues.

The quest for food components that can mitigate adverse metabolic conditions without compromising dietary satisfaction is paramount. By both providing novel data and supporting previous work, our study demonstrates that allulose supplementation in a WD context can modulate body weight, food consumption, and metabolic parameters in a manner that favors improved metabolic health. 

## 5. Conclusions

These findings highlight the potential of allulose as a functional dietary addition that could effectively improve metabolic health outcomes, such as insulin resistance, body weight, and more, all while not forcing potentially difficult dietary changes. Further research is warranted to explore the long-term implications of allulose intake and its application in dietary strategies for the prevention and management of metabolic diseases.

## Data Availability

Data available upon request.
